# Control release of mitochondria-targeted antioxidant by injectable self-assembling peptide hydrogel ameliorated persistent mitochondrial dysfunction and inflammation after acute kidney injury

**DOI:** 10.1080/10717544.2018.1440445

**Published:** 2018-02-16

**Authors:** Meng Zhao, Yijie Zhou, Shuyun Liu, Lan Li, Younan Chen, Jingqiu Cheng, Yanrong Lu, Jingping Liu

**Affiliations:** Key Laboratory of Transplant Engineering and Immunology, Regenerative Medicine Research Center, West China Hospital, Sichuan University, Chengdu, China

**Keywords:** Acute kidney injury, mitochondria, Mito-TEMPO, self-assembling peptide, control release

## Abstract

Persistent mitochondrial injury occurs after acute kidney injury (AKI) and mitochondria-targeted antioxidant Mito-2,2,6,6-tetramethylpiperidine-N-oxyl (TEMPO) (MT) has shown benefits for AKI, but its efficiency is limited by short half-life and side effect *in vivo*. Self-assembling peptide (SAP) hydrogel is a robust platform for drug delivery. This study aims to develop an SAP-based carrier to slow release MT for enhancing its long-term therapeutic potency on AKI. The KLD with aspartic acid (KLDD) was designed. The microstructure and *in vitro* release of MT was assayed. The protective role of MT-loaded SAP (SAP-MT) hydrogel on renal mitochondrial injury, tubular apoptosis, and inflammation was evaluated in mice at five days after ischemia-reperfusion injury (IRI). Our results showed that KLDD could self-assemble into cross-linked nanofiber hydrogel and it had lower release rate than free MT and KLD hydrogel. Compared to IRI and free MT mice, SAP-MT mice exerted reduced renal mitochondria-produced ROS (mtROS) and improved mitochondrial biogenesis and architecture. Consequently, SAP-MT mice showed less renal tubular cell apoptosis, kidney injury marker kidney injury molecule-1 (Kim-1) expression, lower level of pro-inflammatory factors expression, and macrophages infiltration than those of IRI and free MT mice. This study suggested that SAP-MT ameliorated IRI due to its extended mitochondrial protection role than free MT and thus improved the long-term outcomes of AKI.

## Introduction

Acute kidney injury (AKI) is a serious health issue with high morbidity, mortality, and long-term risk of chronic kidney disease (CKD). The common causes of AKI include ischemia, sepsis, and drug-induced nephrotoxicity. Renal ischemia-reperfusion (IRI) is a major reason for AKI (Lameire et al., 2013). Currently, continuous renal replacement therapy (CRRT) is the preferred method to manage fluid overload and metabolic disorders in AKI patients, but it has no direct role to promote kidney recovery after AKI (Thadhani et al., 1996). Many pharmacologic therapies such as diuretics, vasoactive drugs, and growth factors have been widely studied in animal models or preliminary clinical experiment, but their long-term renal outcome has not been revealed by clinic practices. Therefore, novel efficiency therapeutics for AKI remains needed.

Despite the mechanisms of AKI are complex and still remain controversial, oxidative stress has been recognized as a key contributor for ischemic AKI (Himmelfarb et al., 2004). Mitochondria are major sources of intracellular reactive oxygen species (ROS) and mitochondria-produced ROS (mtROS) play an important role in ischemic diseases (Drose & Brandt, 2012; Che et al., 2014). In normal condition, mitochondria are equipped with intrinsic antioxidant mechanism to maintain the intracellular redox homeostasis (Stallons et al., 2013), while stress such as ischemia-reperfusion (IR) caused persistent mitochondrial injury and imbalance of mtROS generation in kidney (Funk & Schnellmann, 2012). The increased mtROS could disturb renal mitochondrial dynamics and energy biogenesis and could also induce tubular apoptosis and necrosis after IR injury (Loor et al., 2011; Che et al., 2014). Therefore, targeting mtROS has been proposed as a potential strategy for treatment of ischemic kidney diseases and recent studies showed the mitochondria-targeted antioxidants have shown promise in recent preclinical studies of AKI (Szeto, 2017).

Mito-TEMPO (MT) is a common mitochondria-targeted antioxidant that consists of 2,2,6,6-tetramethylpiperidine-N-oxyl (TEMPO) and triphenylphosphonium cation (TPP^+^) (Trnka et al., 2008). The TPP^+^ can promote MT pass through the phospholipid bilayer of mitochondria and thus selectively scavenge mtROS by TEMPO (Liang et al., 2010). Previous studies reported that MT could attenuate renal mitochondrial dysfunction in sepsis-induced AKI (Patil et al., 2014) and suppressed the expression of p53 and the ratio of Bax/Bcl2 in ischemic skeletal muscles (Miura et al., 2017). However, the therapeutic efficiency of MT was largely limited by short half-life and side effects *in vivo*. It had been reported that MT-enhanced MRI signals were decreased at 15 min after intravenous injection in mice, which reflected the rapid clearance of MT *in vivo* (Zhelev et al., 2013). Moreover, higher dose of MT showed no renal protective role in AKI mice (Patil et al., 2014), because the accumulated TPP^+^ could depolarize the mitochondria (Trnka et al., 2008). Therefore, development of carriers to slow release MT may reduce its side effect and improve its therapeutic efficiency on AKI.

The injectable hydrogels are of particular interest for drug delivery because they allow for facile injection directly to the target site with subsequent controlled release of encapsulated therapeutics (Lu et al., 2012). Previous study had reported that local delivery of interleukin-10 (IL-10) via injectable HA hydrogels could ameliorate chronic kidney injury (Rodell et al., 2015) as well as ischemic AKI (Soranno et al., 2016) in rodent models. Self-assembling peptide (SAP) was made of natural amino acids and could spontaneously change into hydrogel under physiological saline condition without additional hazard compounds or UV, thus it rarely induced any toxic and immune response for clinical therapies (Liu et al., 2011). Previous reports had proved that SAP was a robust carrier to delivery various drugs and cells *in vitro* and *in vivo* (Zhao & Zhang, 2007; Liu & Zhao, 2011; Liu et al., 2011). The release rates of molecules in SAP hydrogel are affected by the molecular size of drugs, the density of SAP nanofibers, as well as the specific type and number of charged amino acids (Nagai et al., 2006; Gelain et al., 2010). We previously designed SAP with cationic arginine (R^+^) and found that the cationic SAP prolonged the release time of negatively charged hepatocyte growth factor (HGF) and thus enhanced the survival and function of β-cells after transplantation (Liu et al., 2016). These studies suggested that modified SAP with anionic amino acids that could interact with the TTP^+^ might improve the release profile of MT.

In this study, we designed an anionic peptide by addition of aspartic acid (D^−^) into the C-terminal of KLD peptide (KLDD) and assayed the *in vitro* release of MT in KLDD hydrogel. We also evaluated the protective role of MT-loaded KLDD hydrogel on the renal injury, mitochondria function, cell apoptosis, and inflammation in a mice model of IRI.

## Materials and methods

### Preparation of self-assembling peptide

Self-assembling peptides (SAPs) including KLD (n-KLDLKLDLKLDL-c) and KLDD (n-KLDLKLDLKLDLD-c) were commercially synthesized by Shanghai Biotech Bioscience & Technology Co., Ltd (Shanghai, China). All peptides were purified to higher than 95% by hgh-performance liquid chromatography (HPLC; Waters 600E, Waters Corporation, Milford, MA) and their molecular weight (MW) were measured by mass-spectrometer (LCQ Deca XP Max, Thermo Scientific, Waltham, MA). Lyophilized peptides were dissolved at 10 mg/ml in sterile water and used as stock solution.

### Observation of SAP microstructure by transmission electron microscopy (TEM)

The morphologic image was observed using TEM, H-600, Hitachi, Ltd., Japan. Briefly, 10 μl of peptide solution was evenly placed on a carbon-coated copper grid. About 1 min later, the peptide loaded grid was rinsed with Milli-Q water to remove unattached peptide and then negatively stained with 2% phosphotungstic acid solution. After air-drying it in room temperature, the grids were observed by TEM.

### Determination of SAP nanofiber diameter by dynamic light scattering (DLS)

The average particle size of SAP were assayed by DLS using a particle size analyzer (Zetasizer Nano S, Malvern, UK) at room temperature with appropriate viscosity and refractive index settings. SAP stock solution (1%) was diluted with Milli-Q water, and each sample was tested three times to generate the intensity-based size distribution plot report.

### *In vitro* release test of MT from hydrogel

The drug release assay were analyzed by the following procedure: 50 μl of SAP gel containing 5 μl MT (10 mM, Santa Cruz, CA) was placed in the bottom of Eppendorf tube, and then 250 μl of phosphate buffered saline (PBS; 0.1 M, pH =7.2) was added gently and incubated at 37 °C. At the indicated time points, the supernatant was collected and replaced with fresh PBS buffer. The concentration of MT was measured by UV spectrophotometer (Shimadzu, Japan) at 268 nm.

### Induction of kidney IRI model in mice

Eight- to 10-week-old wild-type C57BL/6 male mice (25–30 g) were purchased from Experimental Animal Center of Sichuan University (Chengdu, China). All animal experiments were approved by the Animal Care and Use Committee of Sichuan University and were conducted according to the National Institutes of Health Guide for the Care and Use of Laboratory Animals. Animals were housed in standardized conditions with controlled temperature, humidity, and 12 h cycles of light and darkness, fed with standard chow and tap water *ad libitum*. Mice were anesthetized with 1% pentobarbital sodium (Merck, Germany) solution and underwent bilateral renal ischemia-reperfusion with clamp time of 30 min as previously described (Andres-Hernando et al., 2014). After all surgical procedures, fascia and skin were closed in two layers.

### Kidney free MT and MT-hydrogel injection

Mice were randomly divided into four groups: control, IRI, IRI + free MT, and IRI + MT-loaded SAP (SAP-MT). At 15 min post reperfusion, 25 μl of free MT (0.2 μM) or SAP-MT hydrogel (5 mg/ml KLDD, 0.2 μM MT) were injected at one site under both renal capsules of mice by insulin syringe as previously described (Soranno et al., 2016) and IRI mice were injected with PBS. Five days post surgery, mice were sacrificed by over-dose of 1% pentobarbital sodium, serum and kidney samples including cortex and medulla were collected for further analysis.

### Kidney mitochondrial biogenesis genes assay

Total RNA was extracted from fresh kidney tissue by Trizol (Gibco, Life Technologies, CA) and then reverse-transcribed into complementary DNA (cDNA) by iScript cDNA synthesis kit (Bio-Rad, Hercules, CA). Real-time polymerase chain reaction (Real time-PCR) was performed on a CFX96 real-time PCR detection system (Bio-Rad,) with SYBR Green (Bio-Rad). Primers used in this study are listed in the Supplementary Table S1. The data were analyzed by Bio-Rad CFX Manager software and the relative change of mRNA was calculated by delta-delta Ct method with GAPDH as internal reference gene.

### Measurement of kidney mitochondrial ROS

The mitochondrial ROS was measured by MitoSOX Red (Invitrogen, Invitrogen, Carlsbad, CA), a highly selective fluorescent probe for the detection of oxygen generated within mitochondria. The fresh renal tissues removed from mice were immediately cut into 5 μm thick frozen sections. The renal sections were incubated with 5 μM MitoSox Red in a dark and humidified container at 37 °C for 30 min, and the images were acquired by a fluorescence microscope (Imager Z2, Zeiss, Germany).

### Observation of kidney mitochondrial morphology by TEM

Fresh renal tissues from mice were fixed in 2.5% glutaraldehyde solution and then dehydrated and embedded in EPON resin. Ultra-thin sections of embedded tissues were stained with 5% uranyl acetate and lead citrate solution and were analyzed by TEM.

### Evaluation of kidney injury by histological examination

Kidneys were fixed in 10% formalin and embedded into paraffin, 5 μm sections of embedded kidneys were stained with hematoxylin and eosin (H&E). The cell apoptosis in frozen kidney sections was measured by Terminal deoxynucleotidyl transferase (TdT) dUTP Nick-End Labeling (TUNEL) staining (Promega, Madison, WI) according to the manufacturer’s protocol. The number of TUNEL-positive nuclei per field was evaluated in five fields per section and three sections per kidney. For immunohistochemical (IHC) staining, kidney sections were incubated with primary antibodies including: anti-CD68 (1:100, Abcam, Cambridge, MA), anti- cytochrome C oxidase subunit IV (COX IV; 1:100, Abclonal, USA), anti- mitochondrial transcription factor A (TFAM;1:100, Abclonal) overnight at 4 °C, and then were also incubated with horse radish peroxide-conjugated secondary antibodies (Millipore, Billerica, MA) and 3,3-diaminobenzidine (DAB) substrate. The micrographs of stained sections were captured by light microscopy (Zeiss Imager A2, Germany) and quantified by Image J (NIH, MD, USA).

### Measurement of kidney proteins by immunofluorescence staining (IF)

Frozen tissue sections of kidney from mice were fixed with 4% paraformaldehyde in PBS for 10 min at room temperature, later were washed with PBS and were permeabilized with 0.3% Triton X-100 for 10 min. After blocking in 1% bovine serum albumin for 30 min, the slides were incubated with anti-kidney injury molecule-1 (Kim-1;1:150, R&D Systems, Minneapolis, MN), anti-ICAM-1 (1:100, Proteintech Group Inc., Rosemont, IL), anti-TNF-α (1:100, CST, Boston, MA), anti-TFAM (1:100, Abclonal, Woburn, MA) antibody overnight at 4 °C followed and were by incubation with isothiocyanate (FITC) and tetramethylrhodamine isothiocyanate (TRITC) conjugated secondary antibody for 1 h at 37 °C. Nuclei were visualized by staining with 4',6-diamidino-2-phenylindole (DAPI; Sigma-Aldrich, St. Louis, MO). Digital images were captured by fluorescent microscope (Imager Z2, Zeiss, Germany). Kidney tissue analysis was limited to the renal cortex region.

### Statistical analysis

All data were presented as mean ± SD and analyzed by SPSS software (version 11.5, IBM Corporation, Chicago, IL,) with one-way analysis of variance (ANOVA) test and *p* < .05 was considered as significant difference.

## Results

### Slow release of MT by anionic KLDD hydrogel

**Figure 1.**
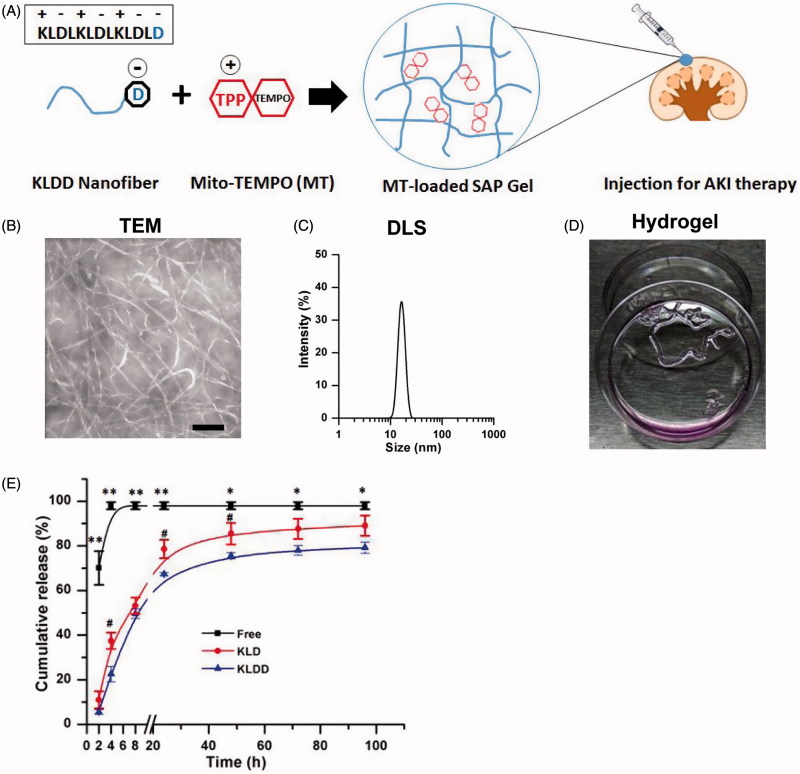
Design and characterization of cationic KLDD peptide. (A) Fabrication of MT-loaded SAP hydrogel for AKI therapy. (B) Representative TEM images of KLDD nanofibers (1 mg/ml, scale bar =100 nm). (C) Determination of the diameter of KLDD nanofibers by DLS. (D) Evaluation of injectable KLDD hydrogel formation in DMEM solution. (E) *In vitro* release of free MT, MT-loaded KLD, and MT-loaded KLDD in PBS (KLDD vs. control, *p < .05; **p < .01; KLDD vs. KLD, #*p* < .05).

The purity and molecular weight (MW) of KLD and KLDD was 95.2/97.8% and 1467.7/1582.8, respectively (Supplementary Figure S1). KLDD had similar properties to the KLD peptide (Supplementary Figure S2), whose self-assembly formed cross-linked nanofibers in aqueous solution (Figure 1(A,B)) with diameters of 10–20 nm (Figure 1(C)). Moreover, KLDD rapidly changed into a hydrogel when injecting into the cell culture media (Figure 1(D), Supplementary Video S1). *In vitro* release results showed that the release rate of MT in KLDD hydrogel was significantly lower than free MT group at all time points. Compared with KLD group, KLDD group also had significant slower release rate at 8 (37.45 ± 3.64 vs. 22.55 ± 3.48, *p* < .05), 24 (78.61 ± 4.17 vs. 67.27 ± 1.69, *p* < .05), and 48 (84.45 ± 4.87 vs. 75.59 ± 1.39, *p* < .05), respectively (Figure 1(E)).

### Slow release of MT rescued persistent renal mitochondrial injury after AKI

**Figure 2.**
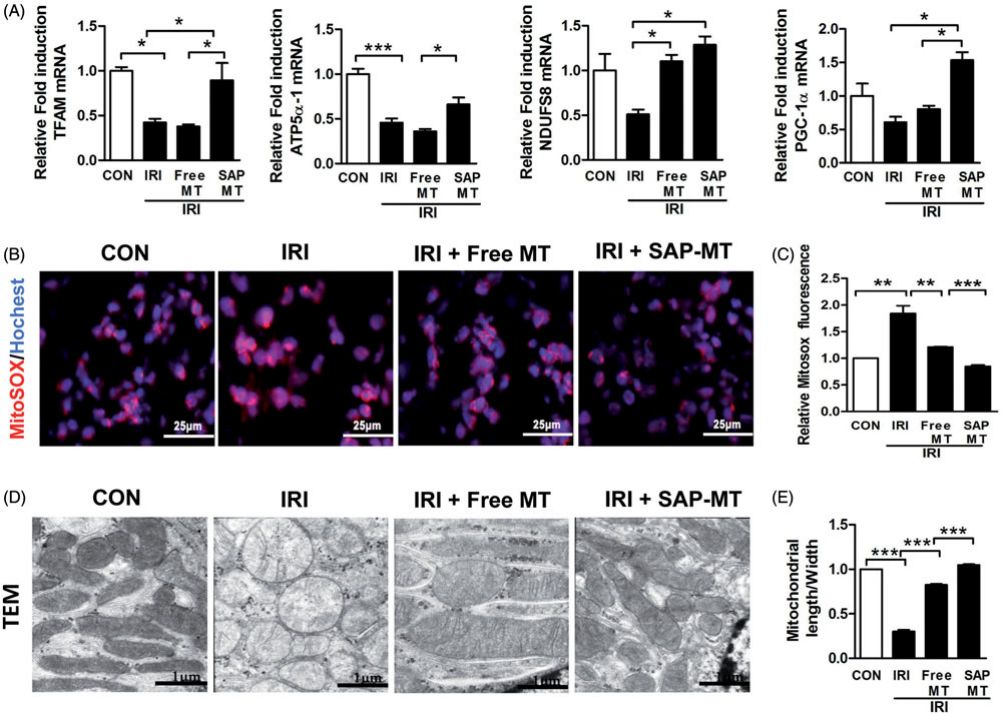
Slow release of MT rescued persistent mitochondria dysfunction after AKI. (A) Real-time PCR analysis for renal mitochondrial function-related genes including TFAM, ATP5α-1, PGC-1α, and NDUFS8 at day 5 after IRI. (B) Detection of kidney mtROS production by frozen section and MitoSOX staining (Scale bar =25 μm). (C) Quantification of mtROS level in kidney measured by MitoSOX staining. (D) Representative TEM micrographs of mitochondria in TECs at day 5 after IRI (Scale bar =1 μm). (E) Quantification of mitochondrial length/width ratio detected by TEM (*p < 005; **p < .01; and ***p < .001).

**Figure 3.**
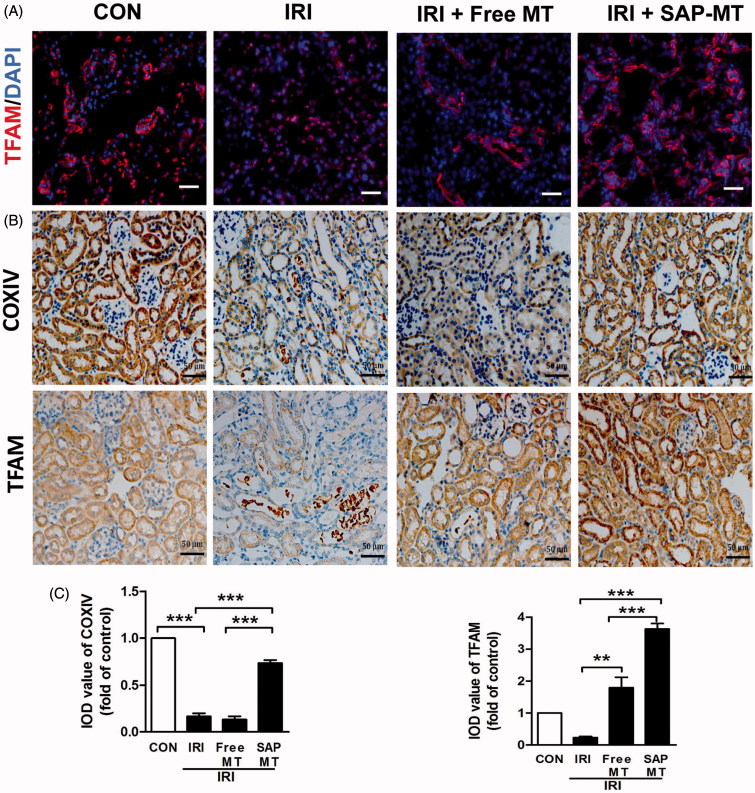
Slow release of MT sustainably restored mitochondria biogenesis after AKI. (A) Representative IF staining for mitochondrial TFAM at day 5 after IRI (Scale bar =50 μm). (B) Representative IHC staining for mitochondria protein COX IV and TFAM at day 5 after IRI (Scale bar =50 μm). (C) Quantification of renal COXIV and TFAM expression detected by IHC using image pro-plus analysis (*p < .05 and **p < .01; ***p < .001).

Compared with controls, IRI mice showed declined renal mitochondrial biogenesis-related genes including Peroxisome proliferator-activated receptor gamma coactivator 1-alpha (PGC-1α; 1.0 ± 0.18 vs. 0.6 ± 0.08), ATP5a-1 (1.0 ± 0.06 vs. 0.46 ± 0.04, *p* < .001), NADH-ubiquinone oxidoreductase(NDUFS8;1.0 ± 0.18 vs. 0.51 ± 0.05), and TFAM (1.0 ± 0.04 vs. 0.42 ± 0.04, *p* < .05; Figure 2(A)) as well as increased mtROS (1.84 ± 0.25 vs. 1.0 ± 0.00, *p* < .01; Figure 2(B,C)), and free MT slightly reduced these mitochondria injuries, whereas SAP-MT significantly reduced mtROS (0.85 ± 0.04 vs. Free MT: 1.21 ± 0.01, *p* < .001) and enhanced PGC-1α (1.54 ± 0.12 vs. Free MT: 0.81 ± 0.05, *p* < .05), ATP5a-1 (0.66 ± 0.07 vs. Free MT: 0.34 ± 0.03, *p* < .05), and TFAM (0.89 ± 0.19 vs. Free MT: 0.38 ± 0.02, *p* < .05) genes expression in kidney of IRI mice (Figure 2(A–C)). TEM results showed mitochondria swelling with reduced matrix density and disrupted cristae architecture in kidney of IRI mice at five days after IRI, while mitochondria in SAP-MT group were elongated with dense cristae membranes and increased length/width ratio (1.05 ± 0.02 vs. Free MT: 0.82 ± 0.02, *p* < .001) compared to IRI and free MT group (Figure 2(D,E)). Furthermore, SAP-MT group showed higher level of renal mitochondria proteins including COX IV (0.73 ± 0.06 vs. Free MT: 0.13 ± 0.06, *p* < .001), and TFAM expression (3.63 ± 0.29 vs. Free MT: 1.80 ± 0.56, *p* < .01) than those of IRI and free MT group (Figure 3(A–C)).

### Slow release of MT alleviated prolonged kidney injury in mice after AKI

**Figure 4.**
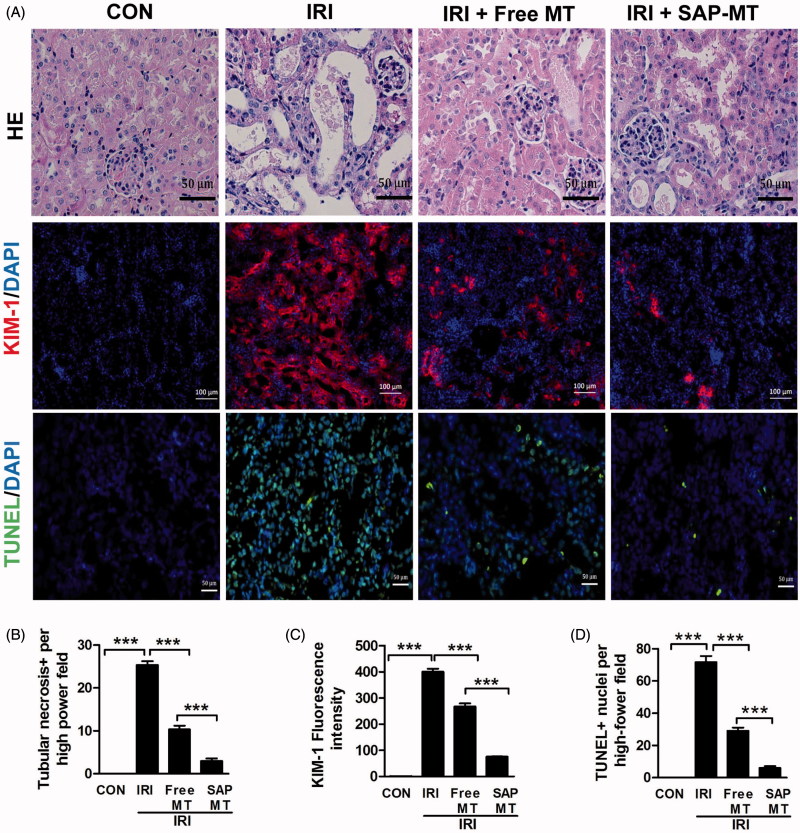
Slow release of MT alleviated prolonged kidney injury after AKI. (A) Representative micrographs of renal HE, TUNEL (Scale bar =50 μm), and Kim-1 IF staining at day 5 after IRI (Scale bar =100 μm). (B) Quantification of tubular necrosis areas detected by HE staining. (C) Quantification of Kim-1 expression in kidney. (D) Quantification of TUNEL-positive cells in kidney (*p < .05; **p < .01 and ***p < .001).

Our results showed that SAP-MT treatment had superior renal protective efficiency than free MT in IRI mice. Compared with controls, IRI mice showed obvious renal tubular necrosis (25.33 ± 1.53 vs. 0.00, *p* < .001) and some hyaline casts in tubules (Figure 4(A,B)  and elevated kidney injury marker Kim-1 expression (400.19 ± 20.12 vs. 1.0 ± 0.00, *p* < .001; Figure 4(A,C))  in the kidney even at five days post IRI. By contrast, SAP-MT treatment significantly alleviated these renal injury marker Kim-1 expression (76.02 ± 2.43 vs. Free MT: 267.30 ± 21.30, *p* < .001) in mice after IRI (Figure 4(A-D)). Moreover, TUNEL staining showed that SAP-MT group had less number of renal apoptotic cells (6 ± 2.43 vs. Free MT: 29.00 ± 3.61, *p* < .001) than IRI and free MT group (Figure 4(A,D)).

### Slow release of MT reduced chronic renal inflammatory response after AKI

**Figure 5.**
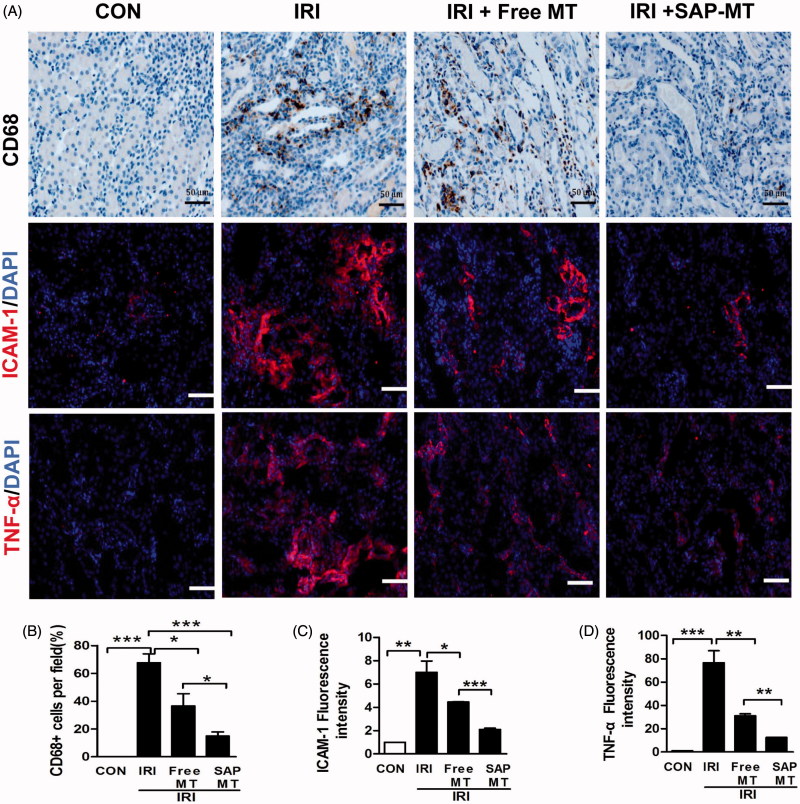
Slow release of MT reduced chronic renal inflammatory response after AKI. (A) Representative micrographs of IHC staining for CD68, ICAM-1, and TNF-α at day 5 after IRI (Scale bar =50 μm). Quantification of (B) CD68-positive macrophages, (C) ICAM-1, and (D) TNF-α protein levels detected by IHC using image pro-plus analysis (*p < .05; **p < .01 and ***p < 0.001).

IRI mice showed increased renal number of CD68^+^ microphages (67.67 ± 10.97% vs. 0.00%, *p* < .001), as well as the expression of pro-inflammatory cytokines including intercellular Adhesion Molecule 1 (ICAM-1;6.99 ± 1.70 vs. 1.0 ± 0.00, *p* < .01) and Tumor necrosis factor-α (TNF-α;76.57 ± 17.93 vs. 1.0 ± 0.00, *p* < .001) compared to control mice (Figure 5(A–D)). By contrast, SAP-MT significantly reduced the pro-inflammatory ICAM-1 expression (2.10 ± 0.2 vs. Free MT: 4.45 ± 0.06, *p* < .001), CD68^+^ microphages infiltration (15.00 ± 5% vs. Free MT: 36.67 ± 15.14%, *p* < .05), and TNF-α protein level (12.54 ± 0.21 vs. Free MT: 31.08 ± 2.82, *p* < .001) in IRI mice, and it showed higher anti-inflammatory effect than free MT group (Figure 5(A–D)).

## Discussion

Increasing evidence indicated that mitochondrial injury played an important role in ischemia AKI (Hall &Schuh, 2016). Since kidney tubular epithelial cells (TECs) were highly rich in mitochondria, IRI inhibited mitochondrial respiration and induced complex electron leak with increased mtROS formation, which could trigger a number of cellular events such as cardiolipin peroxidation, recruiting Bax to mitochondrial permeability transition and cytochrome c release, and further led to inflammasome activation, apoptosis, and tubular necrosis (Iyer et al., 2009). By contrast, mitochondria target antioxidants such as MT could directly scavenge mtROS and indirectly stimulate antioxidative enzymes, thus can serve as potential therapy for AKI (Patil et al., 2014). However, the efficacy of MT was largely limited by its rapid clearance and the potential cytotoxicity due to the TPP^+^, might disturb the mitochondria function and even worsen the kidney injury at higher doses. Therefore, local control release of MT will reduce its side effect and improve its therapeutic potency on kidney injury.

Injective self-assembling peptide hydrogel is a type of amino acid-based biocompatible nanomaterial, which has been used as a robust drug carrier to deliver bioactive compounds and cytokines for diverse applications (Davis et al., 2006; Liu & Zhao, 2011; Liu et al., 2011; Zhao & Zhang, 2006). For instance, SAP hydrogel could effectively extend the release time and the anti-tumor effect of encapsulated paclitaxel *in vitro* (Liu et al., 2011), deliver IGF to reduce the implanted cardiomyocyte apoptosis, and promote myocardial infarction repair *in vivo* (Davis et al., 2006). Previous study had showed that modification of SAP with specific charged amino acids was an efficient strategy to tune the release rates of molecules in SAP hydrogel (Nagai et al., 2006). Because aspartic acid (Asp or D) usually presents negative charge under physiological conditions, we conjunct Asp to the C-terminal of KLD (KLDD) and aim to enhance the binding of MT to SAP nanofibers via the electrostatic interaction between Asp^-^ and TPP^+^. Our results showed that KLDD rapidly changed into nanofiber hydrogel *in situ* when injected in the physiological saline environment without additional chemical compounds, heating, or UV irradiation. Moreover, the *in vitro* release rate of MT in KLDD hydrogel was lower than that of free MT and also significantly slower than KLD hydrogel at several time points. Thus, our data suggested that KLDD hydrogel was a potential carrier to slow release of MT *in vivo*.

Although the extended release of MT in KLDD hydrogel was confirmed *in vitro*, but the effect of slow release of MT on AKI has not been assayed before. IRI was a common cause of AKI with intricate pathological mechanisms such as oxidative stress, mitochondrial injury, and inflammatory response (Zuk & Bonventre, 2016). Oxidative stress upon IR caused direct injury to mitochondria and led to ATP depletion, inflammasome release, and apoptotic cell death after IRI (Loor et al., 2011; Bhargava & Schnellmann, 2017). Thus, we first evaluated the protective role of MT-loaded SAP hydrogel on renal mitochondria injury in IRI mice. PGC-1α was a master regulator of mitochondrial biogenesis and is known to associate with transcription factors responsible for mitochondrial genes such as TFAM expression. Our results showed that IRI mice had increased renal mtROS, mitochondrial fragments, and decline of key mitochondrial biogenesis-related genes such as ATP5a-1, PGC-1α, and TFAM at five days post AKI. These results were highly similar with previous report, which observed renal mitochondria damage even at 2.5 months in AKI rats (Szeto et al., 2017). However, free MT slightly reduced renal mitochondria injury and only improved a few parameters such as mitochondrial complex I subunits (NDUFS8) expression. In contrast, SAP-MT significantly reduced renal mtROS production, enhanced mitochondrial architecture, and almost biogenesis-related genes expression in IRI mice. The possible reason was that single injected free MT was rapidly eliminated *in vivo* and thus could not provide sustained protection as SAP-MT on renal mitochondria after IRI.

The increased mtROS could trigger intracellular pro-apoptotic signals by causing the release of cytochrome c and further led to cell apoptosis and death in renal tubules (Tabara et al., 2014; Chevalier 2016; Duann & Lin, 2017). Kim-1 was a transmembrane protein expressed in damaged tubules and served as a sensitive biomarker for kidney injury (Charlton et al., 2014). Our data showed that SAP-MT significantly reduced acute renal lesions including tubular apoptosis, necrosis, and up-regulation of Kim-1 in IRI mice and it exerted better renal protective effect on AKI than free MT group at five days after IRI. The poor renal protective efficacy of free MT might be due to targeting of only the acute oxidative stress but failure of reduction prolonged damage after IRI. Furthermore, ROS-mediated mitochondrial injury would release damage-associated molecular pattern molecules (DAMPs) that activated the sterile inflammatory response, which in turn contributed to chronic kidney inflammatory injury for days to weeks after AKI (Chen & Nunez, 2010; Arslan et al., 2011). Our data showed that SAP-MT had higher anti-inflammatory role than that of free MT group in IRI mice, which significantly reduced pro-inflammatory cytokines such as ICAM-1 and TNF-α release and macrophages infiltration in kidney at five days after IRI. Taken together, our results suggested that SAP-MT effectively ameliorated cell apoptosis, tubular lesions, and inflammation after IRI due to its extended mitochondrial protection role than free MT, which could target both primary oxidative stress and chronic tubular injury after IRI. Therefore, local slow release of MT by injectable SAP hydrogel was a promising therapy to enhance renal recovery after AKI.

## Conclusions

In summary, our designed injectable KLDD nanofiber hydrogel could prolong the release of MT *in vitro* and thus provided sustained protective role on IRI-induced mitochondria dysfunction in the kidney of mice, which consequently ameliorated the renal tubular lesions and inflammation after IRI. This study suggested that SAP hydrogel served as a promising therapeutic drug carrier to promote kidney repair after renal injury.

## Supplementary Material

IDRD_Liu_et_al_Supplement_Content.doc
