# Supplementary material for: Control release of mitochondria-targeted antioxidant by injectable self-assembling peptide hydrogel ameliorated persistent mitochondrial dysfunction and inflammation after acute kidney injury
Source: Drug Deliv. 2018 Feb 16;25(1):546–54. doi: 10.1080/10717544.2018.1440445 (PMC6058479; doi:10.1080/10717544.2018.1440445)
Supplement: IDRD_Liu_et_al_Supplement_Content.doc [file IDRD_A_1440445_SM2377.doc]

**Supplementary materials**


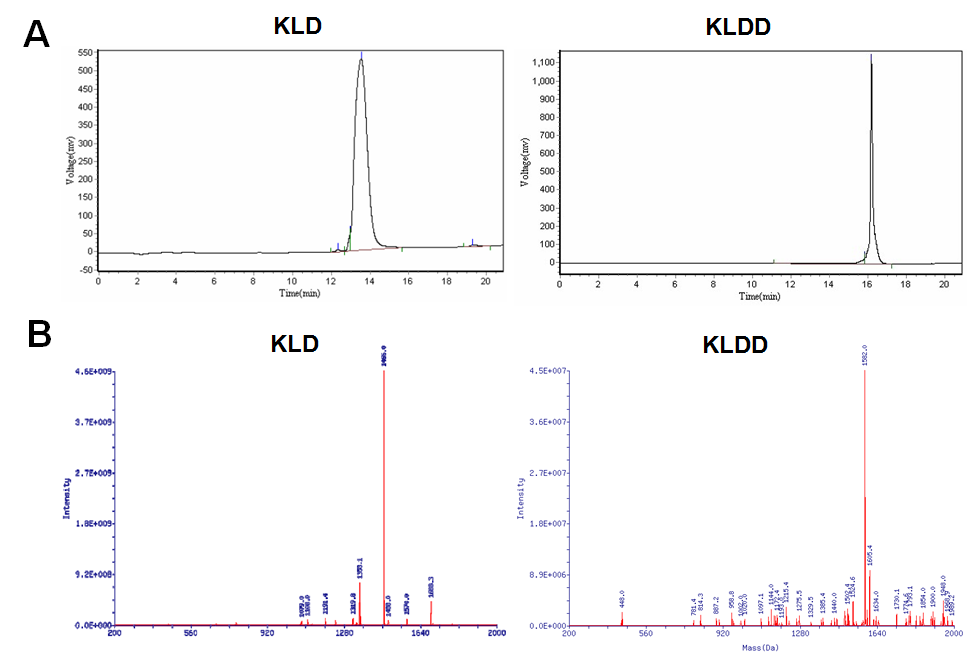
**Figure S1** (A) Measurement the purity of KLD and KLDD peptides by HPLC. (B) Determination the molecular weight (MW) of KLD and KLDD by HPLC-MS.

**
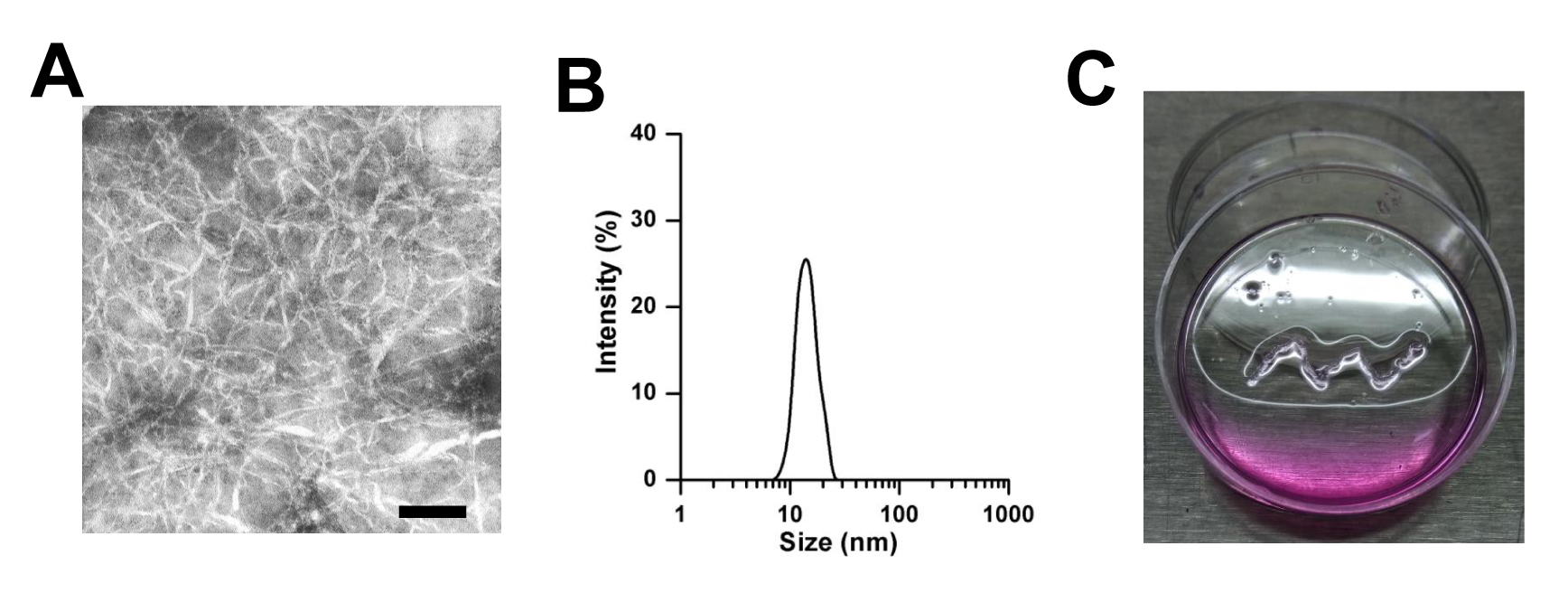
**

**Figure S2** Characterization of KLD peptide. (A) Representative TEM images of KLD nanofibers (1 mg/ml, scale bar = 100 nm). (B) Determination of the diameter of KLD nanofibers by DLS. (C) Evaluation of KLD hydrogel (5 mg/ml) formation in DMEM solution.

**Table S1**

| **Gene** | **Primer sequence** |
| --- | --- |
| PGC-1α (mouse) | Forward: 5′-CACCAAACCCACAGAAAACAG-3′ |
|  | Reverse: 5′-GGGTCAGAGGAAGAGATAAAGTTG-3′ |
| TFAM (mouse) | Forward: 5′- CTGTGGAGGGAGCTACCAGA-3′ |
|  | Reverse: 5′-CCCATCAGCTGACTTGGAGT-3′ |
| NDUFS8 (mouse) | Forward: 5′-AGCCTGCCACCATCAACTAC-3′ |
|  | Reverse: 5′-CACAGAGTTTGCAGGCAATG-3′ |
| ATP5a1 (mouse) | Forward: 5′-CATTGGTGATGGTATTGCGC-3′ |
|  | Reverse: 5′-TCCCAAACACGACAACTCC-3′ |
